# Depression and suicidal behavior among adolescents living with HIV in Botswana: a cross-sectional study

**DOI:** 10.1186/s13034-022-00492-9

**Published:** 2022-07-29

**Authors:** Anthony A. Olashore, Saeeda Paruk, Ontibile Tshume, Bonginkosi Chiliza

**Affiliations:** 1 Department of Psychiatry, Faculty of Medicine, University of Botswana, Gaborone, Botswana; 2 Department of Psychiatry, Nelson R Mandela School of Medicine, University of KwaZulu-Natal, Durban, South Africa; 3 Botswana-Baylor Children’s Clinical Centre of Excellence, Gaborone, Botswana

**Keywords:** HIV, Adolescents, Predictors, Depression, Suicidal behavior, Botswana

## Abstract

**Background:**

Depression and suicidal behavior are the main causes of disability and morbidity, especially in adolescents living with HIV (ALWHIV). Data regarding these are lacking in Botswana, a country with a predominantly youthful population and ranked among the top four in the world most affected by HIV. Therefore, the present study aimed to estimate the prevalence of depression and suicidal behavior and explore their associated factors in Botswana ALWHIV.

**Methods:**

Responses were obtained from 622 ALWHIV using the DSM-5 and the Mini-International Neuropsychiatric Interview for Children and Adolescents.

**Results:**

The mean age (SD) of the participants was 17.7 (1.60) years and more males (54.3%) participated than females. Depression and suicidal behavior rates among adolescents were 23% and 18.9%, respectively. Female participants were more likely to be depressed (AOR = 1.96; 95% CI 1.11–3.45) and have suicidal behaviour (AOR = 6.60; 95% CI 3.19–13.7). Loss of mother (AOR = 2.87; 95% CI 1.08–7.62) and viral load of 400 copies and above (AOR = 5.01; 95% CI 2.86–8.78) were associated with depression. Alcohol use disorder (AOR = 3.82; 95% CI 1.83–7.96) and negative feelings about status (AOR = 8.79; 95% CI 4.62–16.7) were associated with suicidal behavior. Good support (AOR = 0.42; 95% CI 0.23–0.76) and increased frequency of religious activities were protective (AOR = 0.33; 95% CI 0.14–0.79) against depression and suicidal behaviour, respectively.

**Conclusion:**

Therefore, routine psychologic screening, which includes identifying psychological stressors and maladaptive coping, family and caregiver support services, and psychosocial support platforms, should be integrated into the management package for ALWHIV in Botswana.

## Background

Depression is the leading cause of global disability, and it affects approximately 4.4% of the world’s population [1]. Almost one-third of adolescents aged 10–19 years globally are at risk of developing clinical depression, which is higher than the reported estimates for those aged 18 and 25 years [2]. Low and middle-income countries (LMICs), such as those in the Middle East and Africa, have the highest prevalence of clinical depression, and females are more likely to be affected than males [2].

Suicide is the fourth leading cause of death in youth aged 15–29 years old worldwide, with over 700,000 taking their life every year [3, 4]. Africa had the highest rate of adolescents (13–17 years) suicidal behavior from 2003–2015, with a prevalence of 20.4% and suicide planning of 23.7%, according to the Global School-based Student Health Survey (GSHS) data in the LMICs [3].

Depression and suicidal behavior have been described among adolescents living with HIV (ALWHIV); however, studies conducted among these individuals are limited [5, 6]. For example, a systematic review of global studies found only 10 published articles on depression among ALWHIV [6]. This review reported a pool prevalence of 26% (95% CI 18.9–34.8) among ALWHIV, whilst another review of 15 studies in sub-Saharan Africa reported a pooled prevalence rate of 22% (95% CI 12–34) for depression and 11% (95% CI 7–16) for suicidal behavior [7]. Most of these studies are from countries that have made enormous progress in the fight against new HIV infections, while those lagging, such as Ethiopia and Ghana, have limited output regarding the mental health of the ALWIHV [7].

While Botswana, which ranks among the top countries affected by HIV globally, was listed among those that have made remarkable progress in the fight against new infections, ALWHIV still constitute a third of the source of new infections, suggesting more needs to be done [8]. There was a spike in suicidal behavior and depression rates among young people in Botswana in 2021, as shown by the unpublished hospital record in Botswana’s only mental health referral hospital. Studies on depression focused mainly on university students [9–12] and adults in community settings [9–11]. The few studies conducted among individuals living with HIV were mainly in the adult population [13–16] and conducted more than 10 years ago [13, 14]. At the time of writing, no study was found that estimated the prevalence of depression among the ALWHIV in Botswana, which ranked among the top four countries most affected by HIV [12].

Apart from the direct and psychosocial effect of HIV in the development of mental disorders such as depression and suicidal behavior, comorbid depression can worsen treatment and the quality of life in affected individuals [17, 18]. Even though data have shown some rates and correlates of depression and suicidal behavior in the adult population [14, 15], it is inappropriate to assume the same in adolescents who may have different psychosocial needs and stressors. Therefore, the present study intended to fill this gap by estimating the prevalence of depression and suicidal behavior and exploring their associated factors in Botswana ALWHIV.

## Materials and methods

This cross-sectional study involved ALWHIV aged 12–19 years attending HIV clinics, including Botswana Baylor children’s clinical center of excellence (BBCCCE), the regional HIV care clinics in Mahalapye and Lobatse. Most of the participants were recruited from BBCCCE, which is in Gaborone, the capital city of Botswana. Almost 70% of the ALWHIV in the country attend this center, which is managed by a government-private partnership, and provides care for 2404 children, mainly from Gaborone, and over 4000 from other sites in Botswana. Their services include screening, six-monthly viral load checks, medication dispensaries, counseling, research, psychosocial services, education and training, and management of other medical needs of ALWHIV. While other centers are not as comprehensive, they provide similar services close to the residents of ALWHIV.

### Sampling and selection

A minimum sample size of 490 was targeted, with a convenient sampling method adopted, which entailed recruiting willing participants as they came to the clinic until the desired sample was reached. The participants were recruited if they could communicate in English or Setswana, were willing to participate, confirmed to have HIV infection, and were not too physically or mentally ill to participate or follow instructions.

### Study procedure

Five research assistants (RAs), all psychology graduates, were trained to collect data from the adolescents, as well as to administer and score the instruments. All the eligible and willing participants were met on clinic days after their doctors' consultation and briefed on the study procedure. They were interviewed in a private consulting room to observe privacy and confidentiality. All the protocols relating to COVID-19 were observed during the pandemic. The RAs assisted the participants in completing the questionnaire after signing the consent forms to minimize the rate of missing data. Data were collected from November 2019 to December 2021.

A clinic was initiated at the data collection stage and is still running for participants identified as having clinical depression or suicidal ideation and managed by the principal investigator, who is a psychiatrist. Those who required further treatment, especially inpatient care, were referred appropriately without breaching confidentiality.

### Measures

The study questionnaire booklet had three parts: the sociodemographic and clinical part, DSM-5 criteria for alcohol use disorder, and the Mini-International Neuropsychiatric Interview for Children and Adolescents (MINI-KID) modules on depression and suicidal behavior.

The sociodemographic part of the questionnaire included questions about the participant's age, ethnicity, religious participation, parents' marital status, level of education, and occupation. Relevant clinical questions were added and included viral load and frequency of clinic attendance with responses such as (i) Never (ii) Rarely (iii) Sometimes (iv) Frequently (v) Always (vi) Cannot remember. The information in their records was used to corroborate their responses, with frequently and always being coded as ‘good’ attendance while others as ‘poor’. Feelings about HIV status were assessed by asking the participants to express how and what they feel about their status. The responses were transcribed into a) still struggling, having difficulty accepting status, or feels bad about status; b) has accepted status or comfortable living with the status; c) 'I do not know my status but just getting by or just taking the medication because I was told I need it.' Only the first two responses were analyzed.

The Diagnostic and Statistical Manual of Mental Disorders (DSM–5) [19] was used to assess alcohol use disorder (AUD). The DSM-5 combines the categories of alcohol abuse and dependence into a single AUD. It comprises 11 criteria, and only two of them in 12 months are required to diagnose AUD.

The Mini-International Neuropsychiatric Interview for Children and Adolescents (MINI-KID) [20] is a structured, clinical diagnostic interview. The first two modules on depression and suicidality (suicidal behavior) were used in the present study. This tool assesses the presence of ICD-10 and DSM-IV mental disorders comprehensively and concisely. It is an interviewer-administered diagnostic tool, which is suitable for adolescents and been used among ALWHIV in African settings [5, 21]. The outcome variables were depression and suicidal behavior, with the former being present when the diagnostic MINI-KID criteria were met for the current episode. This was used for further analysis, albeit the past episode was also assessed and recorded. The suicidal behavior is scored on a scale and categorically; however, the category ‘YES or NO’ was used for further analysis.

### Data analysis

The data from the current study were analyzed using the Statistical Package for Social Sciences (SPSS for Windows), the 21st version. The mean (SD) was used to describe the continuous variables, such as age at the last birthday and the suicidal behavior score, while percentages were used for the categorical variables, such as religion, religious participation, and gender. The suicidal behavior score was graded and reported as mild, moderate, and severe risks; however, ‘Present or Absent’ (YES or NO) suicidal behavior was used as the outcome. Also, ‘Present or Absent’ was used for depression. All the clinical and sociodemographic variables were entered into a binary logistic regression model to explore the predictors of suicidal behavior and depression in the ALWHIV. The level of statistical significance for all tests was set at p < 0.05.

### Ethical considerations

The Biological Research Ethics Committee of the University of KwaZulu-Natal, University of Botswana Research and Ethical Review Committee (UBIRB), the Ministry of Health and Wellness, and BBCCCE Independent Review Board (IRB) approved this project before starting. The head of all other centers gave their permission to use their facilities. Written informed consent was sought from the participants who were 18 years as at the time of data collection. The parents of the selected ALWHIV provided written consent for those under 18 years. For those who did not come with their parents due to COVID-19, consent was sought telephonically, as recommended by the local IRB.

## Results

### Socio-demographic characteristics of the ALWHIV

Of the 622 participants who completed the study, 338 (54.3%) were males. The mean age (SD) of the participants was 17.7 (1.60) years, and most (60.8%) had junior high school as the highest level of education. Most (64.6%) of the participants were raised by single parents, of which 60.8% were mothers, relatives, family friends, and foster families, while 19.9% had lost both of their parents. A little above half (55.7%) of the participants reported poor social support from their families and friends (Table 1).

**Table 1:** Socio-demographic characteristics of the ALWHIV

Characteristics	Statistic (%)	
Mean age in years (SD)	17.7 (1.60)	
	Frequency N	Percentages
Gender	**622**	**100**
Male	338	54.3
Female	284	45.7
Highest level of education	**612**	**100**
Junior high school and below	372	60.8
Senior High school and above	240	39.2
Religion	**620**	**100**
Christianity	496	80.0
No religion	93	15.0
Others^b^	31	5.0
Type of caregiver	**622**	**100**
Single parent/relatives or others^a^	402	64.6
Both parent	220	35.4
Paternal orphan	**612**	**100**
Yes	137	22.4
No	475	77.6
Maternal orphan	**622**	**100**
Yes	172	27.7
No	450	72.3
Double orphan	**622**	**100**
No	498	80.1
Yes	124	19.9
Perceived social support from family	**618**	**100**
Poor	344	55.7
Good	274	44.3

Total number of the respondents are in bold

^a^family friends and foster family, ^b^Islam, African traditional religion

### Clinical parameters of the ALWHIV

Of the 618 who completed the question on frequency of clinic attendance, 60 (9.7%) reported poor clinic attendance. In addition, about a quarter (26.1%) of the participants had poor viral suppression, as indicated by a viral load of 400 copies and above, which is the Botswana cut-off, and 26.2% reported poor counseling and support from their healthcare providers. One-third (n = 174, 33.8%) reported feeling bad about their HIV status, and 17% reported having drinking problems or AUD (Table 2).

**Table 2:** Clinical parameters of the ALWHIV

Characteristics	Frequency N	Statistic (%)
Clinic attendance	**618**	**100**
Poor	60	9.7
Good	558	90.3
Viral load	**595**	**100**
Below 400 copies	440	73.9
400 copies and above	155	26.1
Support and counselling from care providers	**618**	**100**
Poor	162	26.2
Good	456	73.8
Feeling about HIV status	**574**	**100**
Felt bad about status	194	33.8
Has accepted status	380	66.2
Past episodes of depression	**622**	**100**
Present	99	15.9
Absent	523	84.1
Current episodes	**622**	**100**
Present	147	23.6
Absent	475	76.4
Recurrent episodes	**622**	**100**
Present	45	7.2
Absent	577	92.8
Suicide	**622**	**100**
No risk	505	81.2
Low risk	42	6.8
Moderate risk	42	6.8
Severe risk	33	5.3
Alcohol use disorder	**622**	**100**
Present	110	17.7
Absent	512	82.3

Total number of the respondents are in bold

### Prevalence of depression and suicidal behavior in the ALWHIV

Of the 622 participants, 147 (23.6%) met the criteria for a current depressive episode, 99 (15.9%) had a previous episode of depression, and 45 (7.2%) had a recurrent depressive episode. The past month’s prevalence of suicidal behavior was 117 (18.8%), of which 42 (6.8%) had a mild and moderate risk of suicide, and 33 (5.3%) had a severe risk of committing suicide (Table 2).

### Predictors of depression and suicidal behavior in the ALHIV

Female participants were almost two times more likely to be depressed than their male counterparts (AOR = 1.96; 95% CI 1.11–3.45). Loss of mother (AOR = 2.87; 95% CI 1.08–7.62) and having a viral load of 400 copies and above (AOR = 5.01; 95% CI 2.86–8.78) were significantly associated with depression. Having perceived good support from the healthcare providers (AOR = 0.42; 95% CI 0.23–0.76) and the family, relatives, or friends (AOR = 0.54; 95% CI 0.31–0.94) was shown to be protective (Table 3).

**Table 3:** Logistic regression model showing the predictors of depression in the ALWHIV

Characteristics	Wald	*p*-value	AOR	95% CI	
				Lower	Upper
Gender					
Females	5.41	**0.020**	1.96	1.11	3.45
Age					
Older age	0.66	0.418	0.92	0.75	1.13
Level of education					
Junior high school and below	0.52	0.472	0.82	0.47	1.42
Clinic attendance					
Poor	1.55	0.214	1.79	0.72	4.47
Medication changed more once in 6 mths					
Yes	0.14	0.712	1.11	0.65	1.88
Perceived support from family					
Good	4.71	**0.030**	0.54	0.31	0.94
Support/counselling from health staff					
Good	8.05	**0.005**	0.42	0.23	0.76
Paternal orphan					
Yes	1.19	0.276	0.29	0.03	2.71
Maternal orphan					
Yes	4.49	**0.034**	2.87	1.08	7.62
Double orphan					
Yes	0.49	0.483	0.41	0.04	4.87
Parent marital status					
Single, separated or divorced	0.77	0.381	0.71	0.34	1.5219
Caregiver					
Single parent or others	0.07	0.786	1.10	0.54	2.24
HIV status					
Felt bad about status	0.22	0.640	1.15	0.64	2.05
Viral load					
Below 400 copies	31.7	** < 0.01**	5.01	2.86	8.78
Frequency of religious participation					
Regularly	0.00	0.987	1.01	0.52	1.94
Alcohol use disorder					
Present	0.05	0.824	1.08	0.55	2.13
Having a family member with the same infection					
Yes	0.00	0.997	1.00	0.481	2.09
Religion					
Christianity	0.33	0.567	1.29	0.54	3.08

Significant *p* values in bold

Similarly, female participants were more likely to have suicidal behavior (AOR = 6.60; 95% CI 3.19–13.7). Those who felt bad about HIV status (AOR = 8.79; 95% CI 4.62–16.8) and those who had AUD (AOR = 3.82; 95% CI 1.83–7.96) were more likely to have suicidal behavior. Frequent or regular participation in religious activities was observed to be protective (AOR = 0.33; 95% CI 0.14–0.79) (Table 4).

**Table 4:** Logistic regression model showing the predictors of suicidal behavior in ALWHIV

Characteristics	Wald	*p*-value	AOR	95% CI	
				Lower	Upper
Gender					
Females	25.9	** < 0.01**	6.60	3.19	13.6
Age					
Older age	0.35	0.552	0.93	0.72	1.19
Level of education					
Junior high school and below	1.06	0.300	0.71	0.36	1.37
Clinic attendance					
Poor	0.18	0.670	0.76	0.22	2.68
Medication changed more once in 6 mths					
Yes	0.15	0.695	0.89	0.46	1.67
Perceived support from family					
Poor	1.46	0.227	0.67	0.35	1.29
Support/counselling from health staff					
Poor	2.83	0.092	2.01	0.89	4.51
Paternal orphan					
Yes	0.01	0.933	1.09	0.15	7.89
Maternal orphan					
Yes	0.09	0.769	0.83	0.23	2.93
Double orphan					
Yes	0.11	0.746	1.49	0.13	17.3
Parent marital status					
Single, separated or divorced	0.99	0.321	0.64	0.26	1.55
Caregiver					
Single parent or others	2.37	0.124	1.94	0.83	4.53
HIV status					
Felt bad about status	43.8	** < 0.01**	8.79	4.62	16.7
Viral load					
Below 400 copies	1.03	0.310	1.44	0.72	2.88
Frequency of religious participation					
Regularly	6.16	**0.013**	0.33	0.14	0.79
Alcohol use disorder					
Present	12.7	** < 0.01**	3.82	1.83	7.96
Having a family member with the same infection					
Yes	0.81	0.367	1.46	0.64	3.33
Religion					
Christianity	0.29	0.590	1.33	0.47	3.78

Significant *p* values in bold

## Discussion

The study sought to estimate the prevalence of depression and suicidal behavior in ALWHIV and explore the associated factors. The prevalences of depression and suicidal behavior among adolescents were 23% and 18.9%, respectively. Female participants were more likely to be depressed and have suicidal behavior, with good support and increased religious participation being protective against both.

More than 23% of the ALWHIV in the study reported current depressive episodes. Although this rate was a little lower than the 26% pooled prevalence reported in a global studies review [6], it is consistent with the pooled rate of 22% reported in those from sub-Saharan Africa [7]. In addition, 18.9% had an elevated risk of suicide, which is supported by an earlier study that reported 17% among children and adolescents attending a pediatric clinic in Uganda [22]. Despite the variability in the tools used in measuring these disorders across different settings and geographical regions, depression and the risk of suicide remain high among ALWHIV compared to the general population [23]. These findings thus reiterate the importance of focus on the psychological needs of ALWHIV, as they suffer from similar psychological disorders as the adult living with HIV [6, 7]. Depressive disorders and suicidal behavior may impact HIV disease and quality of life [6, 7], which makes it important to integrate routine screening into the treatment of ALWHIV.

The finding that participants with higher mean scores on suicidal behavior were significantly more likely to be depressed was consistent with the previously documented trend [11, 24], suggesting the need to look for suicidal behavior while screening ALWHIV for depression.

The present study also sought to explore the common predictors of depression and suicidal behavior in the ALWHIV population in Botswana. Gender was significantly associated with depression and suicidal behavior, with females more likely to be depressed and have suicidal behavior than males. Previous literature had reported this pattern in seropositive [6, 7] and seronegative adolescents with depression [2]. The social context in Botswana further supports the preponderance of female ALWHIV reporting more depressive symptoms than males, as they were disproportionally affected by the HIV epidemic. They had increased exposure to stigma, discrimination, forced/transactional sex, gender-based violence, and unplanned pregnancies [8]. Furthermore, although the completed suicide rate was higher among male university students in Botswana, females were more likely to communicate their intentions regarding suicide [11], as in the present study's finding. Therefore, while we continue to heed female clients' needs, effort should be made to actively screen for suicidal behavior in males in a manner that promotes communicating their feelings.

The participants with a higher viral load had more depressive symptoms than those with below 400 copies. Although this present cross-sectional study cannot explore the causal relationship between depression and viral failure, studies have associated HIV viral load with psychological disorders such as depression [25]. For example, a study found an association between depression with increased subsets of CD8 cells, which represent activated CD8 T-lymphocyte, and this may be detrimental to the host's defense against HIV later in the course of the disease [18]. Moreover, as loss of appetite and poor sleep are important symptoms of depression and related to reduced immunity [17], it could be hypothesized that they were responsible for viral failure in ALWHIV. Although this deserves further investigation, the authors proposed that depression negatively impacts HIV disease progression, underscoring the need to routinely screen for depression in ALWHIV.

Significantly, maternally orphaned participants were more likely to have depressive symptoms than those whose mothers were alive. Kim and colleagues [26] associated death in the family with depression among ALWHIV, which may also be the case in Botswana, particularly with the loss of mothers, who not only play a huge role in children’s upbringing and family headship but may be the main household provider in single-parent families [27, 28]. Losing a mother may be traumatic or for some children in Botswana, with most participants being raised by single parents in this study. In Botswana, mothers are generally the primary caregivers, as they usually accompany their children to the health care facilities and oversee their treatment, even though they provide for the family financially [27]. This observation thus emphasizes the need to empower mothers in the fight against new HIV infections in Botswana, specifically among young people [29].

Perceived support from the family and healthcare providers was protective against depression in this sample, as reported in a previous study [30]. Lee and colleagues [31] reported the effect of negative social support on depression, while Goin [30] emphasized the impact of parent involvement in adolescent treatment and support. ALWHIV have increased vulnerability to negative emotions such as catastrophizing, negative rumination, self-blaming, and abnormal emotional responses to adverse life events than their seronegative counterpart [32]. Since these factors are prominent internalizing problems such as depression [33], ALWHIV require active probing, constant reassurance, frequent psychoeducation, and counseling. Therefore, health care providers should pay more attention to the social needs of these individuals in addition to clinical treatment. In addition, parents, and other family members, including friends, should also be educated, empowered, and encouraged to give their support to them.

HIV-infected adolescents who had negative feelings toward their HIV status were eight times more likely to have suicidal behavior. Although studies have not fully explored this relationship in ALWHIV, it is possible that this subgroup found it challenging to adjust to or cope with their status. It may be related to multiple psychosocial issues, including stigma, discrimination, being different from peers, and having to visit clinics or take medication. A review of the literature blamed shame as one of the reasons for poor adjustment to status in the people living with HIV (PLWHIV) [34]. Perhaps the constant thoughts of suicide resulted from seeking a way of escape or maladaptive coping and may also explain the hazardous use of alcohol among the participants.

Alcohol use had been linked to suicidal behavior as PLWHIV with drinking problems were more likely to have suicidal behavior [35]; this finding was replicated in the present study among adolescents. Against the popular belief that alcohol numbs psychological pain, it is a depressant that enhances the prevailing mood and promotes negative thoughts such as hopelessness and suicidal behavior [36]. Furthermore, alcohol lowers inhibition sufficiently for an individual to act on suicidal thoughts. It also inhibits activity in the brain regions such as the prefrontal cortex, caudate nucleus, and subthalamic nucleus, which are responsible for inhibition [37]. Suicide and alcohol are ‘escape coping’ maladaptive strategies. ALWHIV should be routinely screened for these, adequately educated, and trained on better coping strategies.

Conversely, this study identified a potentially adaptive coping style; increased participation in religious activities negatively correlated with suicidal behavior. A study that examined the influence of religious affiliation and suicidal behavior found no relationship between them as in the present study [38]. However, more frequent attendance at religious activities was associated with decreased suicidal behavior scores even after adjusting for social support [38, 39]. Religious coping promotes positive appraisals and may help individuals perceive adverse events such as living with HIV as less stressful. It can also increase social support networks and discourage maladaptive coping such as substance use and ultimately suicidal behavior. Therefore, further studies should explore how religious activity can be integrated into the care plan of ALWHIV.

## Limitations

This study has a number of limitations that may have affected the findings. Some of the data were collected during COVID-19 and may have affected the clinic attendance and thus who was available for participation; hence, it should be cautiously interpreted. It may not be generalizable to the rural settings in Botswana; however, the sample was drawn from the largest center, which serves over 60% of the ALWHIV in the country. In addition, it was the first study to establish the prevalence of depression and suicidal behavior using a rigorous diagnostic tool in the ALWHIV in Botswana.

## Conclusions

As reported in other settings, depression and suicidal behavior rates were high in the ALWHIV in Botswana. Females were more vulnerable than their male counterparts. Those with elevated viral load counts and maternally orphaned ALWHIV were more likely to have depression, while those with AUD who had difficulty adjusting to status had a significant risk of committing suicide. Conversely, family and care provider support protected against depression, whereas increased religious participation appeared protective against suicidal behavior. Therefore, routine psychologic screening, which includes identifying disorders, psychological stressors, and maladaptive coping, should be part of the management package for ALWHIV in Botswana. In addition, integrated HIV care programs such as adolescent-friendly services, family and caregiver support services, and psychosocial support platforms should be implemented.

## Data Availability

The datasets used and analyzed during the current study are available from the corresponding author on reasonable request.
